# Supplementary material for: ssDNA is not superior to dsDNA as long HDR donors for CRISPR-mediated endogenous gene tagging in human diploid RPE1 and HCT116 cells
Source: BMC Genomics. 2023 May 29;24:289. doi: 10.1186/s12864-023-09377-3 (PMC10226222; doi:10.1186/s12864-023-09377-3)
Supplement: Supplementary file 1 — Additional file 1: Figure S1. dsDNA-based endogenous tagging with mNG in HCT116 cells. Figure S2. Validation of the ssDNA production methods. Figure S3. Comparison of Cas12a-mediated knock-in efficiency between dsDNA and ssDNA long donors in HCT116 cells. Figure S4. Analysis of HDR directionality of ssDNA donors using long-read sequencing and knock-knock. Figure S5. Homology-independent integration using mNG donors without HAs. Figure S6. Uncropped images of gels and blots for Figs. 1, 2, S2, and S5. [file 12864_2023_9377_MOESM1_ESM.pdf]

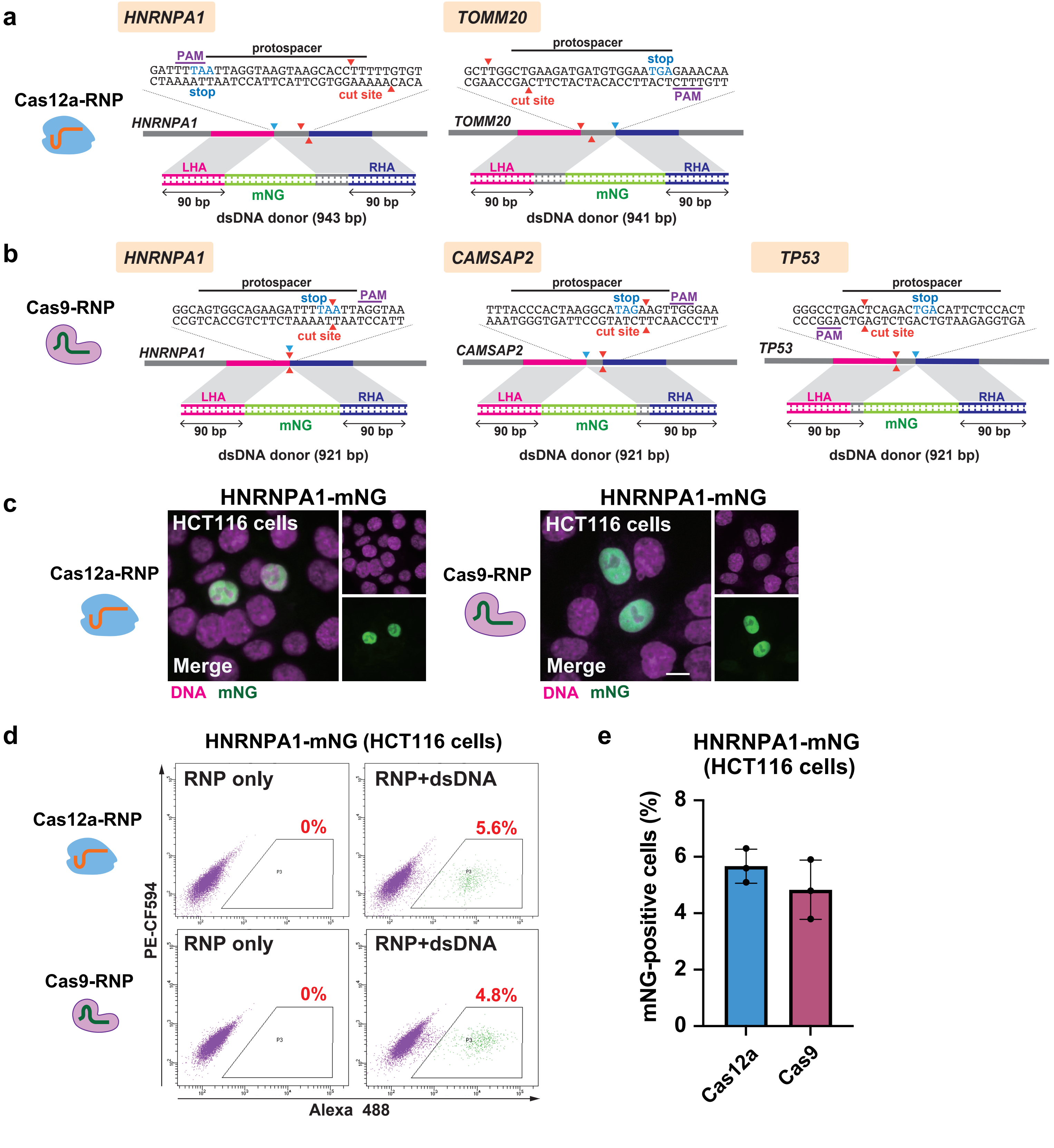

Figure S1

dsDNA-based endogenous tagging with mNG in HCT116 cells.

a, Schematic overview of Cas12a-mediated endogenous tagging of HNRNPA1 and TOMM20 with mNG using dsDNA donors. LHA: left HA, RHA: right HA. **b**, Schematic overview of Cas9-mediated endogenous tagging of HNRNPA1, CAMSAP2, and p53 with mNG using dsDNA donors. LHA: left HA, RHA: right HA. **c**, Representative images of HCT116 cells with Cas12a or Cas9-mediated endogenous mNG tagging of HNRNPA1. Cells at 8 days (Cas12a) or 3 days (Cas9) after electroporation were fixed and analyzed. Scale bar: 10 μm **d**, Flow cytometric analysis of Cas12a or Cas9-mediated HNRNPA1-mNG knock-in in HCT116 cells. Cells at 6 days (Cas12a) or 3 days (Cas9) after electroporation were analyzed. Percentages of cells with mNG signal are shown in the plots. **e**, Quantification of percentages of mNG-positive cells from (**d**). Data from three biological replicates are shown. Approximately 20,000 cells were analyzed for each sample. Data are represented as mean ± S.D.

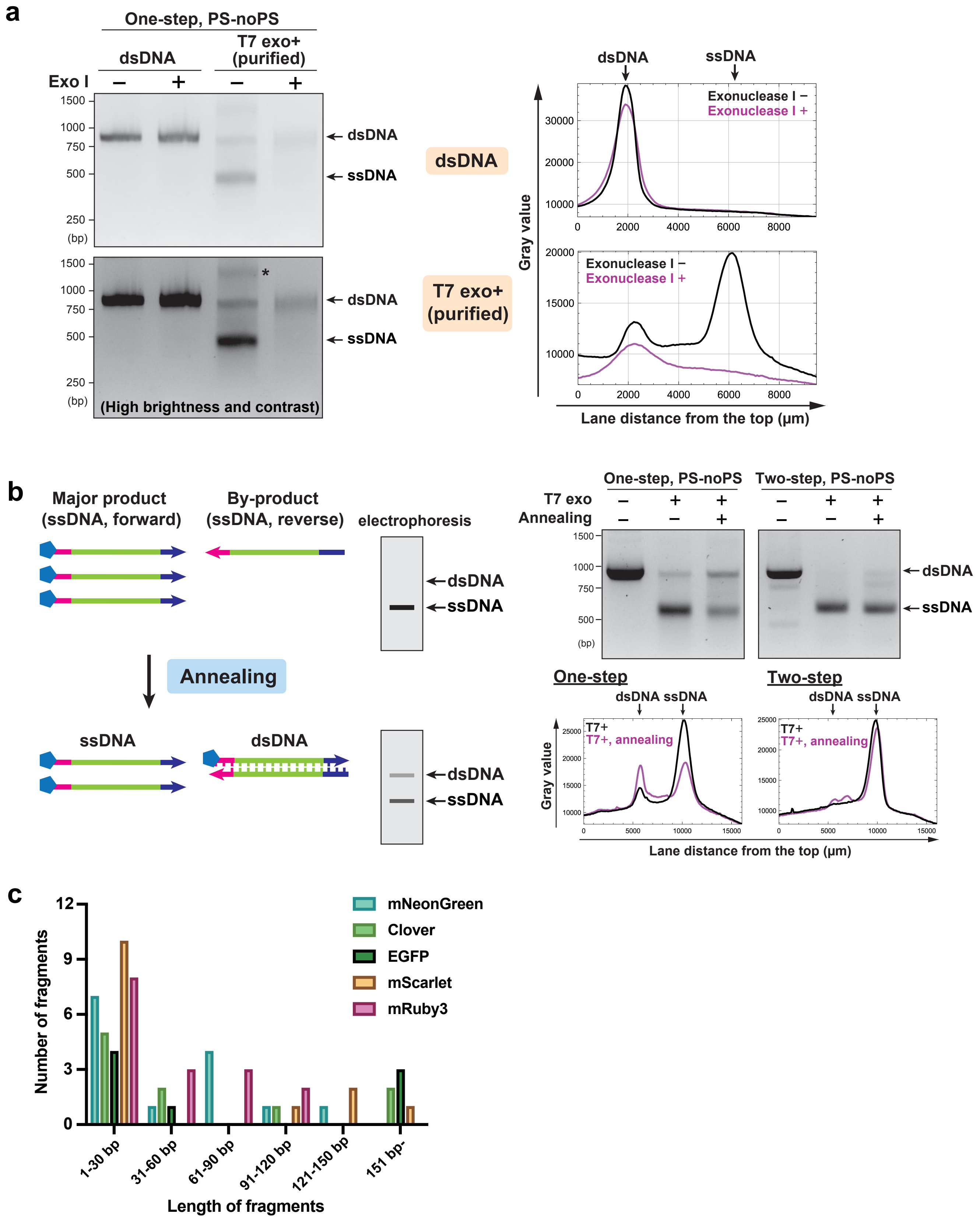

Figure S2

Validation of the ssDNA production methods.

a, Biochemical validation of ssDNA production with ssDNA-specific exonuclease I (Exo I). The indicated DNA products reacted with exonuclease I were analyzed by gel electrophoresis. The DNA sequence of the donor for Cas12a-mediated mNG tagging of HNRNPA1 was used in this experiment. An asterisk shows an ssDNA band derived from non-specific PCR products. The bottom image is of the same gel as the top one, with higher brightness and contrast. Plot profiles for each lane are shown on the right side of the gel electrophoresis image. **b**, (Left panel) Schematic overview of an annealing-based assay for evaluation of the strand selectivity of ssDNA production. When the reverse single strand (by-product) is mixed with the forward single strand (target product), the annealing process results in the production of dsDNA. (Right panel) Gel electrophoresis of DNA products with the indicated reactions. In the condition of the one-step PCR method, the dsDNA band was stronger and the ssDNA band was weaker when annealing was conducted, indicating the contamination of the unintended strand. Plot profiles for each lane are shown below the gel electrophoresis images. The two gel images are cropped from the same gel image. **c**, Histogram showing the frequency of lengths of fragments produced when DNA sequences encoding representative fluorescent reporters are cleaved with a combination of the four restriction enzymes (HpyCH4III, Hpy188I, NlaIII, and RsaI). Full-length gels are presented in Fig. S6.

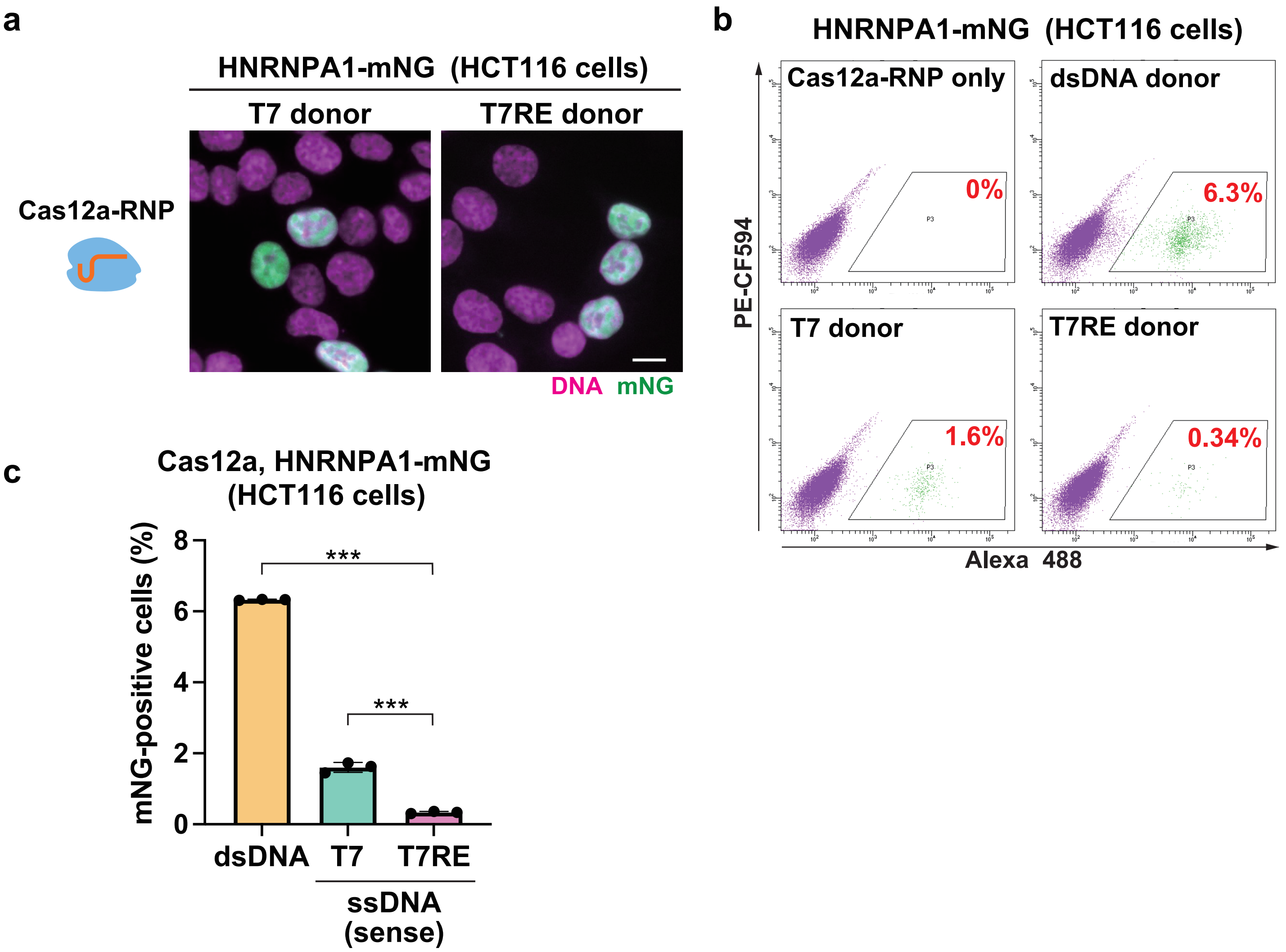

Figure S3

Comparison of Cas12a-mediated knock-in efficiency between dsDNA and ssDNA long donors in HCT116 cells.

a, Representative images of HCT116 cells with Cas12a-mediated mNG tagging of HNRNPA1 using T7 or T7RE donors (sense strands). Cells at 7 days after electroporation were fixed and analyzed. Scale bar: 10 μm. **b**, Flow cytometric analysis of Cas12a-mediated HNRNPA1-mNG knock-in in HCT116 cells, using dsDNA, T7, and T7RE donors at 33 nM. Cells at 5 days after electroporation were analyzed. Percentages of cells with mNG signal are shown in the plots. **c**, Quantification of percentages of mNG-positive cells from (**b**). Data from three biological replicates are shown.

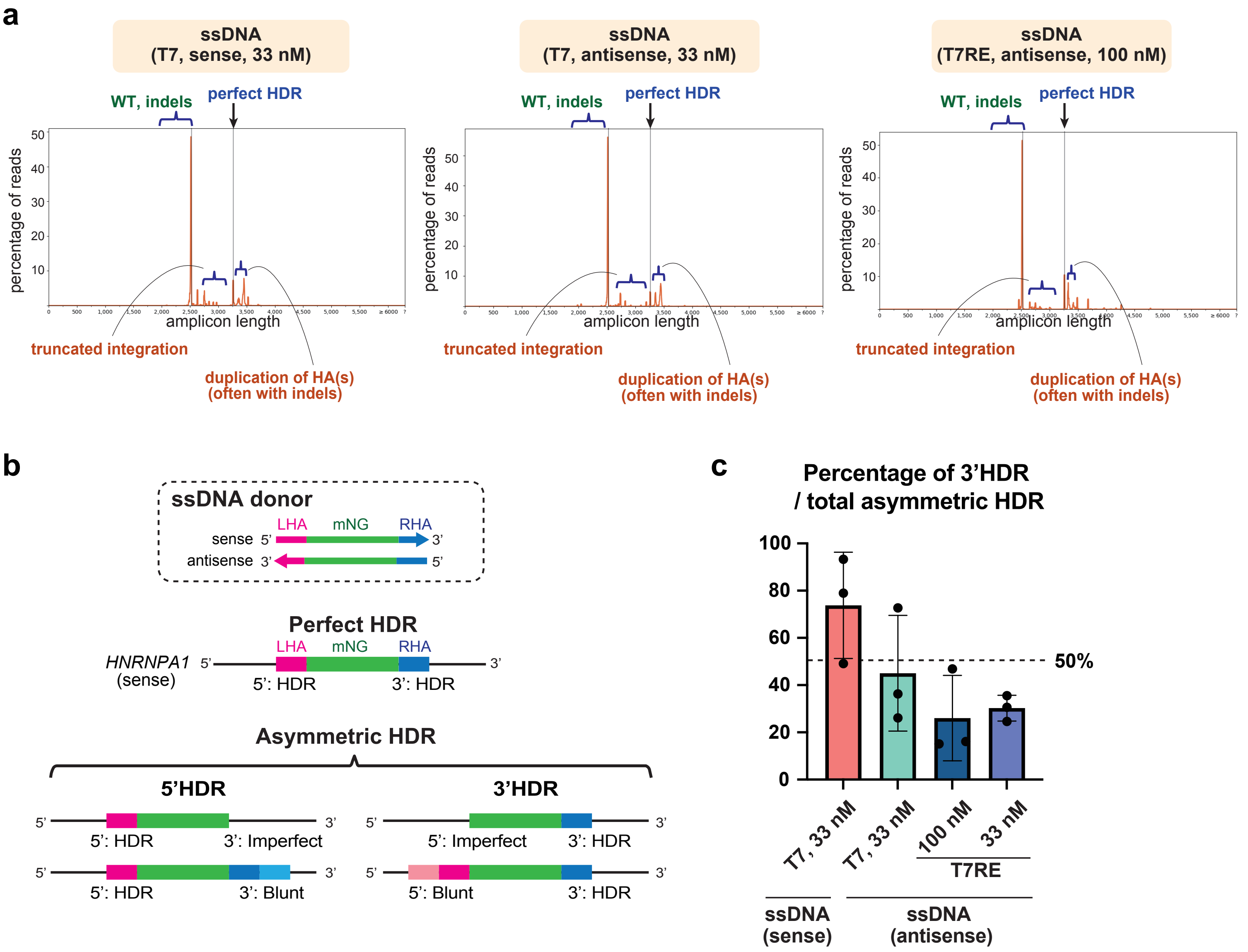

Figure S4

Analysis of HDR directionality of ssDNA donors using long-read sequencing and *knock-knock*.

a, Representative plots generated by knock-knock showing the distribution frequency of amplicon length for conditions other than those shown in Fig. 4b. The range of read lengths corresponding to WT and indels, perfect HDR, truncated integrations, and duplication of homology arm(s) are indicated. **b**, Description of asymmetric HDR, 5' HDR, and 3' HDR. LHA: Left HA, RHA: Right HA. **c**, The frequencies of 3' HDR in the asymmetric HDR events. Data from three biological replicates are shown. Data are presented as mean ± S.D.

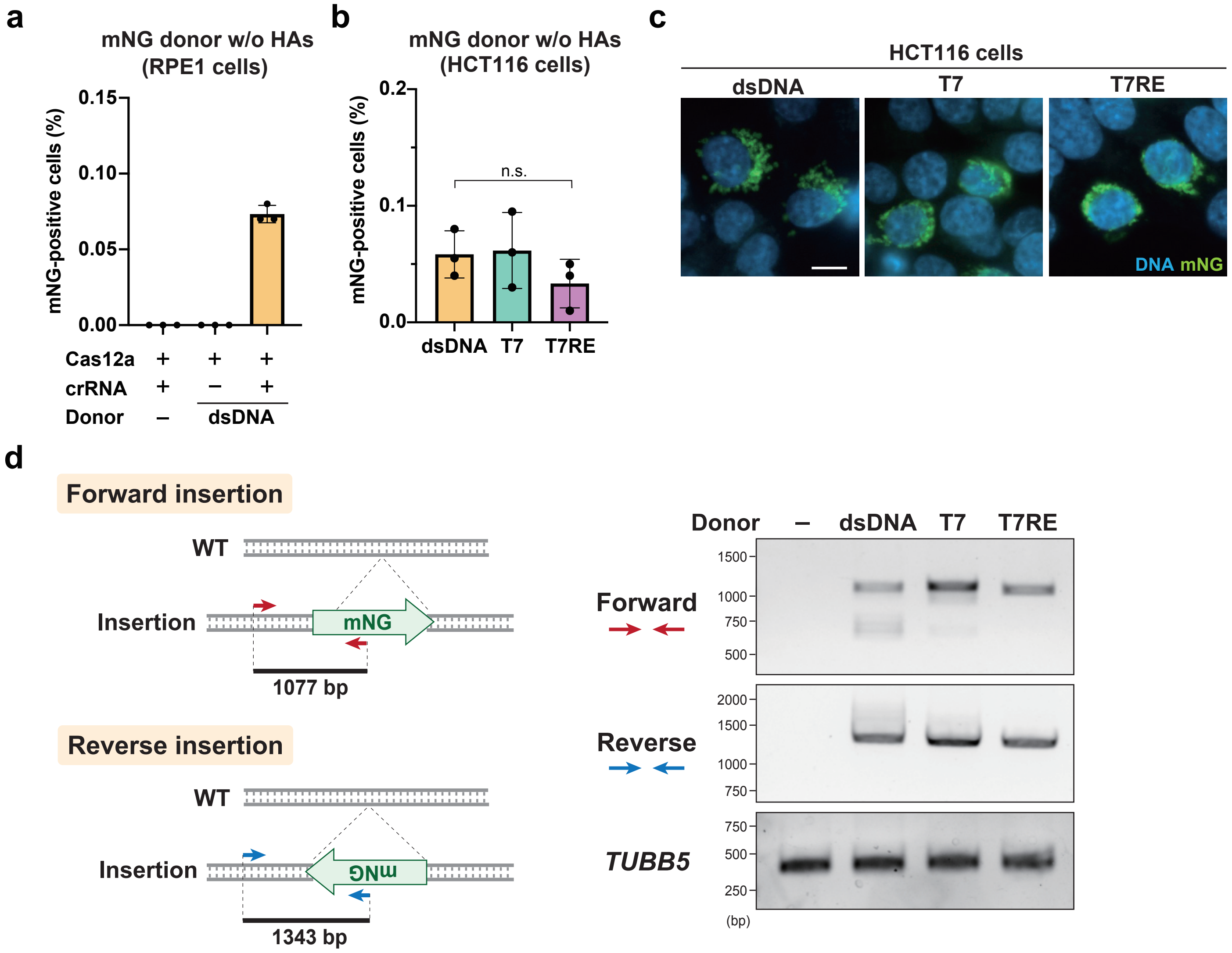

Figure S5

Homology-independent integration using mNG donors without HAs.

a, Flow cytometric analysis of the homology-independent integration of mNG donors into the TOMM20 locus using RPE1 cells. The dsDNA donor concetration was 33 nM. Cells at 7 days after electroporation were analyzed. Data from three biological replicates are shown. 10,000 cells were analyzed for each sample. **b**, Flow cytometric analysis of the homology-independent integration of mNG donors into the TOMM20 locus using HCT116 cells. Sense strands were used for T7 and T7RE donors. All donor concentrations were 33 nM. Cells at 5 days after electroporation were analyzed. Data from three biological replicates are shown. 20,000 cells were analyzed for each sample. A two-tailed, unpaired Student' s t-test was used to obtain the P-value. n.s.: Not significant. **c**, Representative images from the homology-independent integration experiment using HCT116 cells. Cells at 5 days after electroporation were fixed and analyzed. Scale bar: 10 μm. **d**, Genomic PCR detecting the homology-independent insertion of the mNG donors into the TOMM20 locus of RPE1 cells. The two primer sets were designed to amplify the 5' junction of the forward and reverse insertions of the mNG sequence, respectively. *TUBB5*: loading control. Full-length gels are presented in Fig. S6.

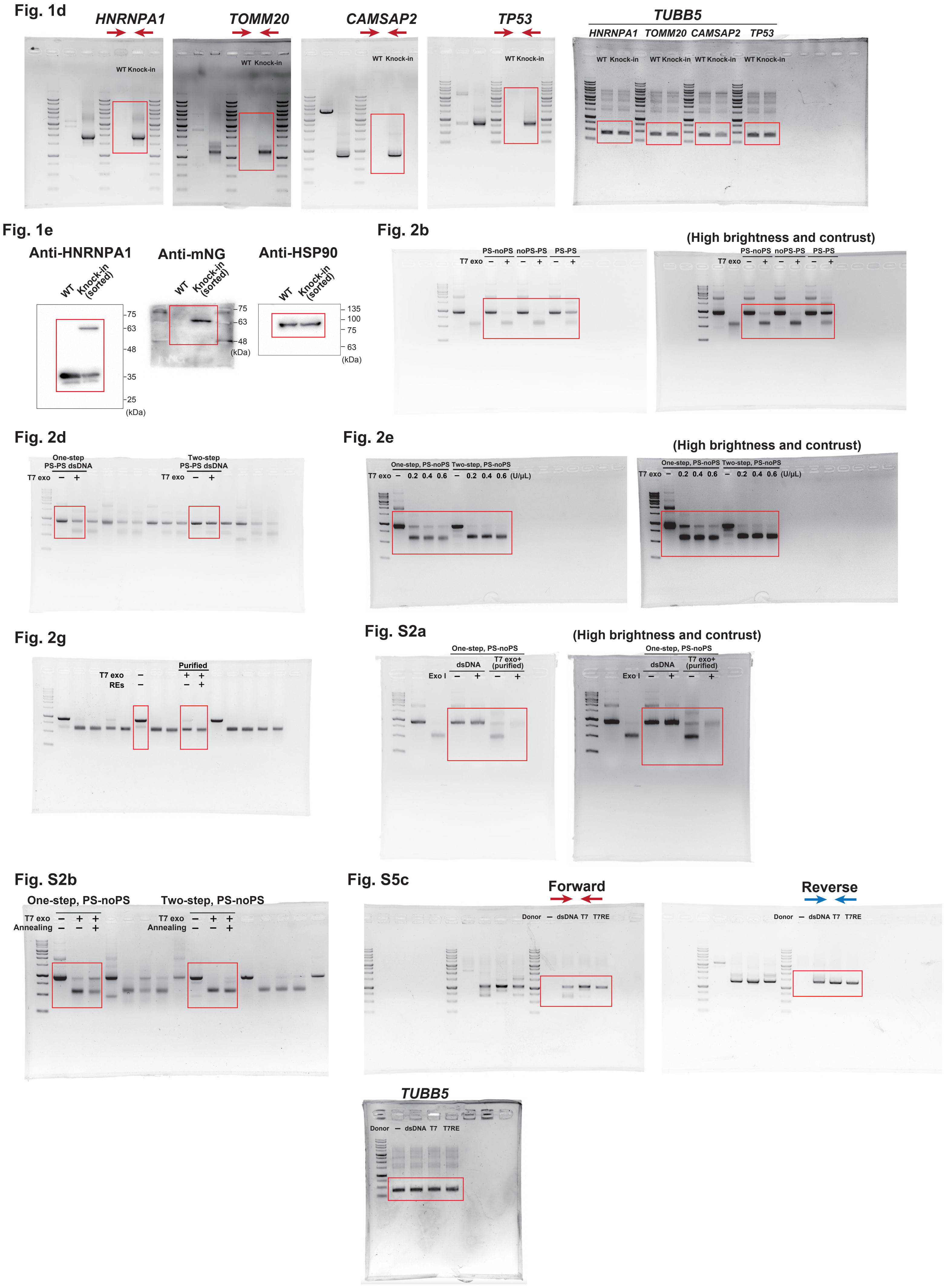

Figure S6 Uncropped images of gels and blots for Fig. 1, 2, S2, and S5.
