# Supplementary material for: ssDNA is not superior to dsDNA as long HDR donors for CRISPR-mediated endogenous gene tagging in human diploid RPE1 and HCT116 cells
Source: BMC Genomics. 2023 May 29;24:289. doi: 10.1186/s12864-023-09377-3 (PMC10226222; doi:10.1186/s12864-023-09377-3)
Supplement: Supplementary file 2 — Additional file 2: Table 1. Primer sequences for PCR. Table 2. Target site sequences of guide RNA. [file 12864_2023_9377_MOESM2_ESM.pdf]

Supplementary Table 1: Primer sequences for PCR

For guide RNA assembly

Name	Sequence
Cas12a_crRNA_Fw	TTCTAATACGACTCACTATAGTAATTTCTACTCTTGTAGAT
HNRNPA1_crRNA_Rv	AAGGTGCTTACTTACCTAATATCTACAAGAGTAGAAATTAC
TOMM20_crRNA_Rv	CTGAAGATGATGTGGAATGAATCTACAAGAGTAGAAATTAC
Univ_sgRNA_Fw	TTCTAATACGACTCACTATAG
Univ_sgRNA_Rv	AAAAGCACCGACTCGGTG
crRNA_tracrRNA	GTTTTAGAGCTAGAAATAGCAAGTTAAAATAAGGCTAGTCCGTTATCAACTTGAAAAAGTGGCACCGAGTCGGTGCTTTT
HNRNPA1_sgRNA_Fw	TTCTAATACGACTCACTATAGAGTGGCAGAAGATTTTAATT
HNRNPA1_sgRNA_ Rv	TTCTAGCTCTAAAACAATTAAAATCTTCTGCCACT
CAMSAP2_sgRNA_Fw	TTCTAATACGACTCACTATAGACCCACTAAGGCATAGAAGT
CAMSAP2_sgRNA_Rv	TTCTAGCTCTAAAACACTTCTATGCCTTAGTGGGT
p53_sgRNA_Fw	TTCTAATACGACTCACTATAGGAGAATGTCAGTCTGAGTC
p53_sgRNA_Rv	TTCTAGCTCTAAAACGACTCAGACTGACATTCTCC

For HDR donor preparation

Name	Sequence
HNRNPA1-mNG_Fw	CACTTTGAAACTTTAAAAGAAAAATTGTACTTTTCAGGTGGCTATGGCGGTTCCAGCAGCAGCAGTAGCTATGGCAGTGGCAGAAGATTTGGAGCTGGTGCAGGTGCAG
HNRNPA1-mNG_Fw_5S	C*A*C*T*T*TGAAACTTTAAAAGAAAAATTGTACTTTTCAGGTGGCTATGGCGGTTCCAGCAGCAGCAGTAGCTATGGCAGTGGCAGAAGATTTGGAGCTGGTGCAGGTGCAG
HNRNPA1-mNG_Rv	ACTGCAATTATAATGTTAACTATGTTGCACTGCTCAGCTACATTAGGGTTATTGGGTTCATCAGCAATTTAAAAAATTATGTCAACACACAAAAAGGTGCTTACTTACCTAACTACTTGTACAGCTCGTCCATGC
HNRNPA1-mNG_Cas12a_Rv_5S	A*C*T*G*C*AATTATAATGTTAACTATGTTGCACTGCTCAGCTACATTAGGGTTATTGGGTTCATCAGCAATTTAAAAAATTATGTCAACACACAAAAAGGTGCTTACTTACCTAACTACTTGTACAGCTCGTCCATGC
HNRNPA1-mNG_2nd_Fw	CACTTTGAAACTTTAAAAGAAAAATTG
HNRNPA1-mNG_2nd_Fw_5S	C*A*C*T*T*TGAAACTTTAAAAGAAAAATTG
HNRNPA1-mNG_Cas12a_2nd_Rv	ACTGCAATTATAATGTTAACTATG
HNRNPA1-mNG_Cas12a_2nd_Rv_5S	A*C*T*G*C*AATTATAATGTTAACTATG
TOMM20-mNG_Fw	TATTTTGAAGTTAGAATCCTAATTAAATGCTTATGACACTTTAAAAAATTATTTTTTTTTCTTTCAGAGAATTGTAAGTGCTCAGAGCTTGGCTGAAGATGATGTGGAAGGAGCTGGTGCAGGTGCAG
TOMM20-mNG_Rv	ATATTTGCCCTTATTCCCCCAGAGCTGCTCAACTACCAAGAATTTTTAAAATATTTTTAACTGAGATTTTATTATGTTGACATTTGTTTCCTACTTGTACAGCTCGTCCATGC
TOMM20-mNG_2nd_Fw_5S	T*A*T*T*T*TGAAGTTAGAATCCTAATTAAATGC
TOMM20-mNG_2nd_Rv	ATATTTGCCCTTATTCCCCCAG
HNRNPA1-mNG_Cas9_Rv	TGTTGCACTGCTCAGCTACATTAGGGTTATTGGGTTCATCAGCAATTTAAAAAATTATGTCAACACACAAAAAGTTGCTTACTTACCTAACTACTTGTACAGCTCGTCCATGC

HNRNPA1-mNG_Cas9_2nd_Rv	TGTTGCACTGCTCAGCTAC
CAMSAP2-mNG_Fw	TCTGCCAGTGTTGATGCAATTACCATTCATAGCCATTTATGGCAGACCAAAAGACCAGTAACACCCAAAAAACTTTTACCCACTAAGGCAGGAGCTGGTGCAGGTGCAG
CAMSAP2-mNG_Rv	TCTTGTTGGCAATTAGAAAGATTTTCTATAGGCAGGAAAGATGAAGTGCAAATTTACCATGAATGTTCTGAAGCAAGTATTTCCCAACTTCTACTTGTACAGCTCGTCCATGC
p53-mNG_Fw	GTCTCCTACAGCCACCTGAAGTCCAAAAAGGGTCAGTCTACCTCCCGCCATAAAAAACTCATGTTCAAGACTGAAGGGCCTGACTCAGACGGAGCTGGTGCAGGTGCAG
p53-mNG_Rv	GCAAGGGTTCAAAGACCCAAAACCCAAAATGGCAGGGGAGGGAGAGATGGGGGTGGGAGGCTGTCAGTGGGGAACAAGAAGTTGAGAATGCTACTTGTACAGCTCGTCCATGC
mNG_Fw	GGAGCTGGTGCAGGTGCAG
mNG_Fw_5S	G*G*A*G*C*TGGTGCAGGTGCAG
mNG_Rv	CTACTTGTACAGCTCGTCCATGC
GalNAcT2_Fw	TCGATCCTCCCTTTATCCAG
GalNAcT2_Fw_5S	T*C*G*A*T*CCTCCCTTTATCCAG
GalNAcT2_Rv	CGGAATTCCCATAGAGCCCA

For genomic PCR and long-read amplicon sequencing

Name	Sequence
HNRNPA1_Up_Fw	CAGGCCTTCAGCCGTTACAC
TOMM20_Up_Fw	TTGGTTTGCCTGGGAGTGAAA
check_mNG_Cas12a_Rv	GCGAGTTGGTCATCACAGGA
TUBB5_Fw	TGTCCCTTTCGTGAACCACC
TUBB5_Rv	ACCCTCCGTTAGATTTCAGAACA
CAMSAP2_Up_Fw	CCCAGTGCAAAATCCAATAAGCA
check_mNG_CAMSAP2_Rv	CCATGTCAAAGTCCACACCGTT
TP53_Up_Fw	GTGACCCCCGTCAAACTCAG
check_mNG_TP53_Rv	TGTCAAAGTCCACACCGTTGA
TOMM20-mNG_woHA-Rv_Fw	GCTGAGGTGCACTTTGAGTTT
TOMM20-mNG_woHA-Rv_Rv	TGAAGAACCAGCCGATGTACG
SMRT_1st_Fw	[AmC6]GCAGTCGAACATGTAGCTGACTCAGGTCACCAGGCCTTCAGCCGTTACAC
SMRT_1st_Rv	[AmC6]TGGATCACTTGTGCAAGCATCACATCGTAGCCCAACCAGAACCCAGTCAAACT

Supplementary Table 2: Target site sequences of guide RNA

Target gene	Cas nuclease	Target sequence
HNRNPA1	Cas12a	ATTAGGTAAGTAAGCACCTT
TOMM20	Cas12a	TCATTCCACATCATCTTCAG
HNRNPA1	Cas9	AGTGGCAGAAGATTTTAATT
TP53	Cas9	GGAGAATGTCAGTCTGAGTC
CAMSAP2	Cas9	ACCCACTAAGGCATAGAAGT
